# Expression and Function of *PML-RARA* in the Hematopoietic Progenitor Cells of *Ctsg-PML-RARA* Mice

**DOI:** 10.1371/journal.pone.0046529

**Published:** 2012-10-08

**Authors:** Lukas D. Wartman, John S. Welch, Geoffrey L. Uy, Jeffery M. Klco, Tamara Lamprecht, Nobish Varghese, Rakesh Nagarajan, Timothy J. Ley

**Affiliations:** 1 Section of Stem Cell Biology, Division of Oncology, Department of Medicine, Washington University School of Medicine, St. Louis, Missouri, United States of America; 2 Pathology and Immunology, Washington University School of Medicine, St. Louis, Missouri, United States of America; Emory University, United States of America

## Abstract

Because *PML-RARA*-induced acute promyelocytic leukemia (APL) is a morphologically differentiated leukemia, many groups have speculated about whether its leukemic cell of origin is a committed myeloid precursor (e.g. a promyelocyte) versus an hematopoietic stem/progenitor cell (HSPC). We originally targeted *PML-RARA* expression with *CTSG* regulatory elements, based on the early observation that this gene was maximally expressed in cells with promyelocyte morphology. Here, we show that both *Ctsg*, and *PML-RARA* targeted to the Ctsg locus (in *Ctsg-PML-RARA* mice), are expressed in the purified KLS cells of these mice (KLS = Kit^+^Lin^−^Sca^+^, which are highly enriched for HSPCs), and this expression results in biological effects in multi-lineage competitive repopulation assays. Further, we demonstrate the transcriptional consequences of *PML-RARA* expression in *Ctsg-PML-RARA* mice in early myeloid development in other myeloid progenitor compartments [common myeloid progenitors (CMPs) and granulocyte/monocyte progenitors (GMPs)], which have a distinct gene expression signature compared to wild-type (WT) mice. Although *PML-RARA* is indeed expressed at high levels in the promyelocytes of *Ctsg-PML-RARA* mice and alters the transcriptional signature of these cells, it does not induce their self-renewal. In sum, these results demonstrate that in the *Ctsg-PML-RARA* mouse model of APL, *PML-RARA* is expressed in and affects the function of multipotent progenitor cells. Finally, since *PML/Pml* is normally expressed in the HSPCs of both humans and mice, and since some human APL samples contain TCR rearrangements and express T lineage genes, we suggest that the very early hematopoietic expression of *PML-RARA* in this mouse model may closely mimic the physiologic expression pattern of *PML-RARA* in human APL patients.

## Introduction

The fusion gene *PML-RARA* is produced by t(15;17)(q22;q21), and is found only in the hematopoietic cells of patients with acute promyelocytic leukemia (APL). When *PML-RARA* is expressed in mice using regulatory elements from the human or mouse cathepsin G gene (*CTSG/Ctsg*) or the human *S100A8 (MRP8)* promoter/enhancer, it can initiate APL; when *RARA* or *PML-RARA* are expressed in mouse bone marrow cells via retroviral transduction, both can decrease myeloid maturation and increase self-renewal [1], [2], [3], [4]. Human APL is associated with differentiation arrest at the promyelocyte stage; in mouse models of the disease, this maturation arrest is less pronounced and varies between models, for reasons that are not yet clear. However, the disease is always myeloid-restricted [5]. Because murine models of APL were designed to target *PML-RARA* expression to myeloid-restricted cells, we and others have suggested that myeloid-restricted disease might result from targeted expression of *PML-RARA* to the promyelocyte compartment [6], [7], [8], [9], [10]. However, human *PML* and murine *Pml* are expressed in early CD34^+^ hematopoietic progenitor cells, and human *PML-RARA* expression may not be limited to committed myeloid progenitors and promyelocytes [11], [12].

Several studies have suggested that in APL, the leukemic cell of origin must be a committed myeloid progenitor [10]. First, Turhan et al. did not observe *PML-RARA* expression in flow-sorted CD34^+^/CD38^−^ cells (a cell population enriched for less mature hematopoietic progenitors, including stem cells), but did detect *PML-RARA* expression in CD34^+^/CD38^+^ cells (a population enriched for more mature hematopoietic progenitors, including early myeloid committed progenitors) from two APL patients, using semi-quantitative RT-PCR [7]. Secondly, Bonnet and Dick observed engraftment of CD34^+^/CD38^−^ AML cells into NOD/SCID mice, but no engraftment of similarly sorted CD34^+^/CD38^−^ cells from APL patients, suggesting that these were not the initiating cells for this subtype of AML [13]. Many authors have suggested that mouse models of APL support this hypothesis, since expression of *PML-RARA* under the control of *Ctsg* or *MRP-8* regulatory elements has led to myeloid leukemia [8], [9], [10], [14]. However, Chapiro *et al.* recently reported expression of T-lineage transcripts and TCR rearrangements in 60% of hypogranular t(15;17) APL cases, suggesting the translocation may affect HSPCs with the capacity to differentiate into both myeloid and lymphoid lineages [14]. In addition, APL cells often do not express CD34 on the cell surface, but do often express atypical lymphoid linage markers (CD56, CD19, or CD2), again suggesting that *PML-RARA* may initiate disease (in human patients) within a multipotent progenitor compartment [15].

In this study, we use state-of-the-art flow-sorting, mRNA amplification, and expression profiling strategies to carefully define the timing of activation of *Ctsg* and *PML-RARA* during early hematopoietic development in *Ctsg-PML-RARA* mice. We found that *Ctsg* mRNA is expressed not only in the KLS (Kit^+^/Lin^−^/Sca^+^) compartment, but also in SLAM cells (CD150^+^/CD41^−^/CD48^−^ KLS), which are even more primitive. We observed striking changes in the gene expression profile of flow-sorted common myeloid progenitors (CMPs) and granulocyte/monocyte progenitors (GMPs) derived from *Ctsg-PML-RARA* mice, which suggests that *PML-RARA* has important transcriptional consequences in early myeloid progenitor cells. We extend these findings with functional validation of *PML-RARA* effects on lymphoid and erythroid lineages, which confirm that *PML-RARA* is expressed (and functional) at a very early stage in the hematopoietic development of *Ctsg-PML-RARA* mice. We observed that in flow-sorted *Ctsg-PML-RARA* promyelocytes, *PML-RARA* expression does result in significant gene expression changes but does not result in distinct gene expression signature, nor does it promote self-renewal. These results change our understanding of the cellular compartments in which *PML-RARA* initiates leukemia in the *Ctsg-PML-RARA* mouse model of APL.

## Results

### Expression of PML-RARA and the genes used to direct expression in APL mouse models

We analyzed the expression profiles of flow-sorted SLAM cells (cKit^+^Lin^−^Sca^+^CD150^+^CD41^−^CD48^−^), KLS cells (cKit^+^Lin^−^Sca^+^), CMPs (Lin^−^Sca-1^−^cKit^+^CD34^+^FcγRII/III^lo^), GMPs (Lin^−^Sca-1^−^cKit^+^CD34^+^FcγRII/III^hi^), megakaryocyte-erythrocyte progenitors (MEPs; Lin^−^Sca-1^−^cKit^+^CD34^−^FcγRII/III^lo^), “promyelocytes"/early myeloid cells (Ly6g^int^SCC^int^B220^−^CD115^−^Ter119^−^), and neutrophils (Ly6g^+^SCC^high^B220^−^CD115^−^Ter119^−^) from 2–6 individual *Ctsg-PML-RARA* or WT mice (Figure S1).

**Figure 1:**
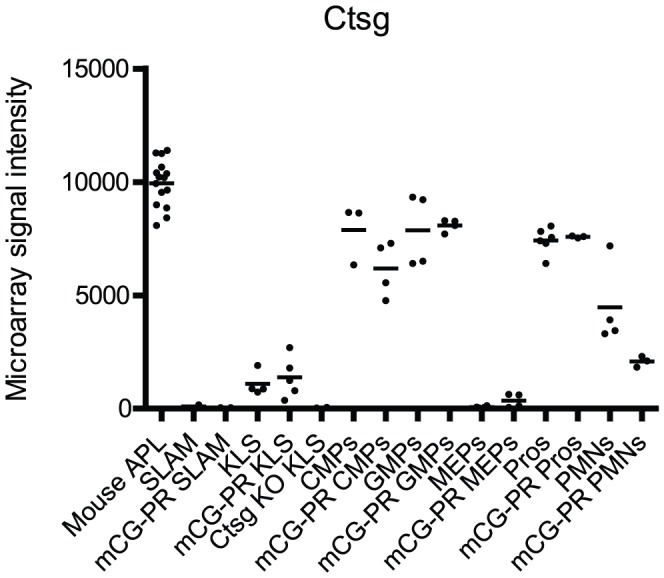
Expression of *Ctsg* in flow-sorted bone marrow cells and mouse leukemia samples. Expression profile in indicated WT and *Ctsg-PML-RARA* (labeled mCG-PR) flow-sorted bone marrow cells and 15 *Ctsg-PML-RARA* leukemia samples (labeled Mouse APL) using Nugen amplified mRNA and Affymetrix Mouse Exon 1.0ST arrays. We plotted *Ctsg* expression for probeset 5542324. Note the absence of *Ctsg* mRNA in *Ctsg* deficient cells (labeled Ctsg KO). There were no statistically significant differences in *Ctsg* expression between the same cell populations, when comparing WT and *Ctsg-PML-RARA* samples (using two-tailed t test, p-value cutoff 0.05). Comparing the levels of Ctsg expression between the *Ctsg-PML-RARA* samples using a two-tailed t test, the p-value for SLAM vs. KLS = 0.11, KLS vs. CMPs = 0.00026, CMPs vs. GMPs = 0.022, GMPs vs. MEPs 2.4×10^−8^, MEPs vs. Pros 2.6×10^−7^, GMPs vs. Pros = 0.027, Pros vs. PMNs = 2.4×10^−6^.

In our mouse model of APL, *PML-RARA* is inserted into the 5′ untranslated region of *Ctsg*, and the *Ctsg* locus therefore regulates its expression [2]. To begin to define when *PML-RARA* expression is activated in this model during hematopoietic development, we first examined the expression of the *Ctsg* gene in all the compartments listed above, using young WT and *Ctsg-PML-RARA* mice (Figure 1). *Ctsg* expression is not consistently detected in the SLAM compartment by exon array. However, KLS cells express detectable amounts of *Ctsg* mRNA by exon array, and this expression is absent in KLS cells derived from *Ctsg* deficient mice (proving the specificity of the probesets for *Ctsg* transcripts). *Ctsg* expression increases massively in the CMP compartment; the high level of expression persists in the GMP compartment and in promyelocytes, and declines in neutrophils. *Ctsg* is minimally expressed in the MEP compartment.

Using very sensitive quantitative RT-PCR to further investigate the timing of *Ctsg* activation, we found that *Ctsg mRNA* could be detected not only in the KLS compartment but also in SLAM cells (Figure S2A). However, we could not reliably detect *PML-RARA* expression in SLAM cells, demonstrating that *PML-RARA* expression is at or below the level of detection by RT-PCR (Figure S2B and S2C). We have previously shown that the expression of the *PML-RARA* transgene driven from the *Ctsg* locus is much lower than that of endogenous *Ctsg* from a WT locus, so this result is consistent with those findings [1], [2]. As expected, *PML-RARA* mRNA was not detected in bone marrow cells from WT littermate controls [8].

We noted that *Ly6g* (*Gr-1*) expression was limited to promyelocytes and neutrophils, and that *Kit* and *Flt3* expression declined during myeloid maturation, as expected (Supplemental Figures S3A, S3B, and S3C). Promyelocytes had relatively low but detectable expression of *Cd34* with an average expression intensity of 785.912±72.575 in *Ctsg-PML-RARA* promyelocytes (n = 3) and 467.255±189.475 in WT promyelocytes (n = 6) with much higher *Cd34* expression in the earlier myeloid compartments, CMPs and GMPs, as expected (Figure S3D). Importantly, we detected no expression of *Ly6g* in SLAM and KLS cells, demonstrating efficient negative selection of differentiated myeloid cells within these populations: all probes had signal intensity <100, whereas signal intensity <200 represents experimental ‘noise’ on this platform (Figure S3A).

**Figure 2:**
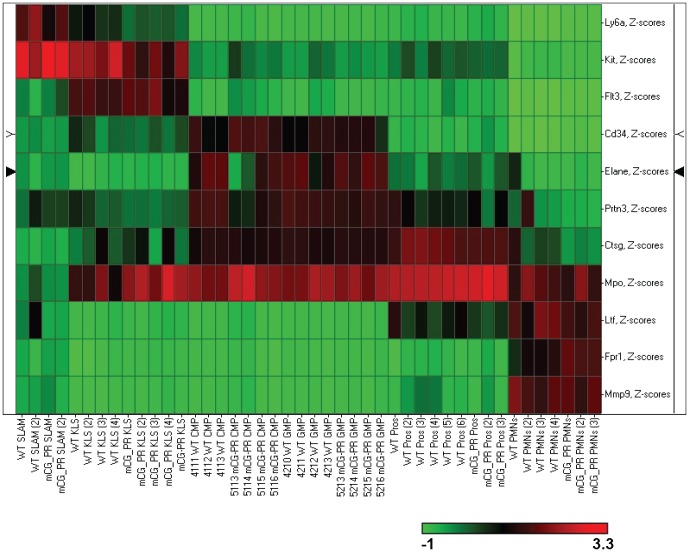
A supervised heatmap of 11 developmentally-regulated myeloid genes. Using the expression data from the Affymetirix Mouse Exon 1.0ST arrays, we created a supervised heatmap with SPOTFIRE using z-score averaging of the probeset with the highest average expression for each of 11 developmentally-regulated myeloid genes across all of our flow-sorted bone marrow cell samples. The legend is shown below the heatmap with downregulated genes in green and upregulated genes in red.

We confirmed the purification of the sorted compartments by plotting the expression of myeloid genes that are known to be developmentally-regulated: myeloperoxidase (*Mpo*), elastase (*Ela2*), and proteinase3 (*Prtn3*) are expressed early in myeloid development in primary granules; lactoferrin (*Ltf*) is expressed later in secondary granules; and matrix metallopeptidase 9 (*Mmp9*) found in tertiary granules is a marker of mature neutrophils (Figure S4A–E). [9], [16], [17]. In addition, we show that the mature myeloid markers formyl peptide receptor 1 (*Fpr1*) and lysozyme-2 (*Lyz2*) are appropriately expressed in our sorted cell populations (Figure S4F and S4G) [18]. The expression data from 11 developmentally-regulated myeloid genes was used to construct a supervised heatmap (by z-score) which clearly illustrates the expected levels of gene expression at the appropriate stage of myeloid development for our sorted cell populations (Figure 2).

**Figure 3:**
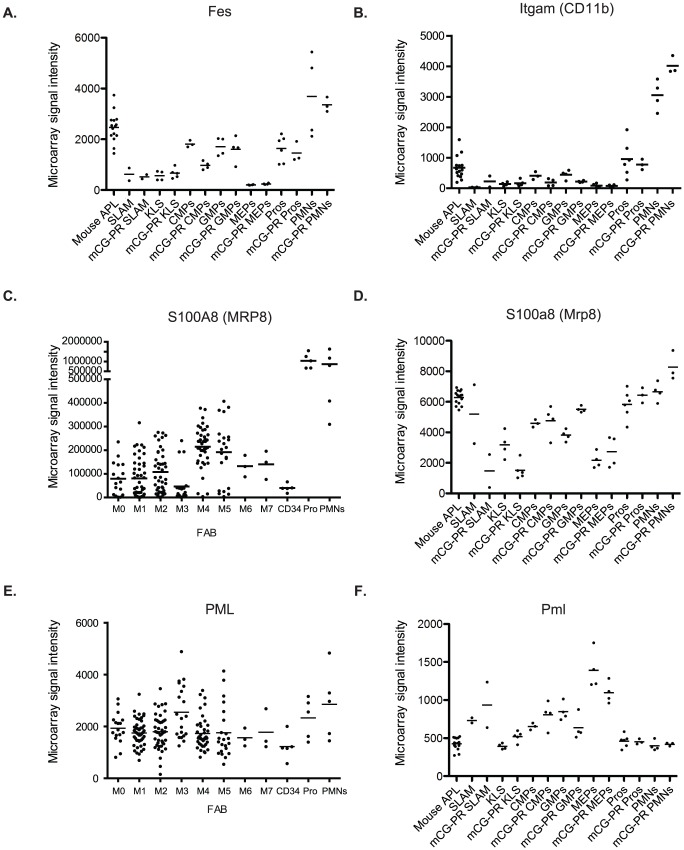
Expression of genes used to target *PML-RARA* expression in mice. Expression data for the indicated genes in flow-sorted bone marrow cells using murine Affymetrix Exon 1.0ST arrays or Human Genome U133 Plus 2.0 arrays. Each panel is a highly representative probe on the array. A. *cFes* (probeset 4758608). B. *Itgam* (CD11b, probeset 4782002). C. *S100A8* (*MRP8*, probeset 202917). D. *S100a8* (*Mrp8*, probeset 5010279). E. *PML* (probeset 235508). F. *Pml* (probeset 4885841). We have previously published the expression profile of *PML* in AML and human hematopoietic cells [11]. Panel E includes an additional 86 AML cases of AML and data from a second representative probe.

Regulatory elements from other “myeloid-restricted" genes have also been used to generate mouse models of APL. One transgenic strategy (using the *S100A8* (MRP8) 5′ flanking region) resulted in myeloid leukemia, while two others (using *Fes* and *Itgam* (CD11b) promoters) did not [3], [19], [20]. Using data from the same expression arrays, we found that *Fes* and *Itgam* are both expressed at much lower levels in KLS cells than *Ctsg*, with expression peaking in neutrophils, rather than promyelocytes (Figure 3A and 3B). In contrast, *S100A8* is highly expressed in human AML cells and in flow-sorted CD34^+^ cells, with massive up-regulation at the promyelocyte stage (Figure 3C). High expression of *S100a8* was also seen in murine hematopoietic cells, although dynamic regulation during promyelocyte maturation was absent (Figure 3D). The expression pattern of these loci contrasts with that of human *PML* and murine *Pml*, which are expressed at much lower levels, and which exhibit little or no dynamic regulation during myeloid maturation (Figure 3E and 3F).

### Effects of Ctsg-PML-RARA in multiple hematopoietic compartments

To determine whether *PML-RARA* expression from the *Ctsg* locus results in biological effects on multipotent hematopoietic cells, we used four independent approaches. First, we used transcriptional profiling using exon arrays of SLAM, KLS, MEP, CMP, GMP, promyelocyte and mature neutrophil populations from *Ctsg-PML-RARA* and WT mice. We performed unsupervised hierarchical clustering analyses to assess the global effects of *PML-RARA* expression on gene expression in KLS and SLAM cells. We observed that KLS (but not SLAM) samples segregated by genotype in an unsupervised clustering analysis, suggesting that expression of *PML-RARA* within the KLS compartment significantly alters the expression of a specific set of genes (Figure S5A). Although many genes were significantly dysregulated in the comparison between WT and *Ctsg-PML-RARA* promyelocytes, this cell population did not cluster by genotype in an unsupervised analysis (Figure S5B).

To assess the downstream consequences of *PML-RARA* expression in early myeloid progenitors, we purified CMPs and GMPs for expression analysis. These populations segregated by *Ctsg-PML-RARA* genotype in unsupervised hierarchical clustering, whereas MEPs did not cluster by genotype (Figure S6). To ascertain significant differences in specific gene expression between the *Ctsg-PML-RARA* and WT samples, we performed ANOVA and Significance of Microarrays (SAM) analysis to define significantly dysregulated genes in each subset (Tables S1, S2, S3, S4, S5, S6, S7, S8, S9, S10, S11, S12, 13 and Data S1). In the KLS comparison, one of the most significantly dysregulated genes by ANOVA was *Notch1* (Table S2), which is relevant for the recent finding of activated Notch1 signaling in both human and murine APL pathogenesis (Grieselhuber NR et al., submitted). Unexpectedly, one of the most significantly dysregulated genes within the CMP and GMP compartments was *Cadherin 1* (*Cdh1*), a tumor suppressor in epithelial cancers, which was strikingly up-regulated in both the CMP and GMP compartments of *Ctsg-PML-RARA* mice (Table S7) [21]. We also found that *cyclin H* (*Ccnh*) expression is down-regulated in the CMP compartment of *Ctsg-PML-RARA* mice (Table S3): cyclin H has been previously shown to be part of a complex that phosphorylates the AF-2 domain of RARA, leading to the downstream activation of retinoic acid responsive genes [22].

**Figure 4:**
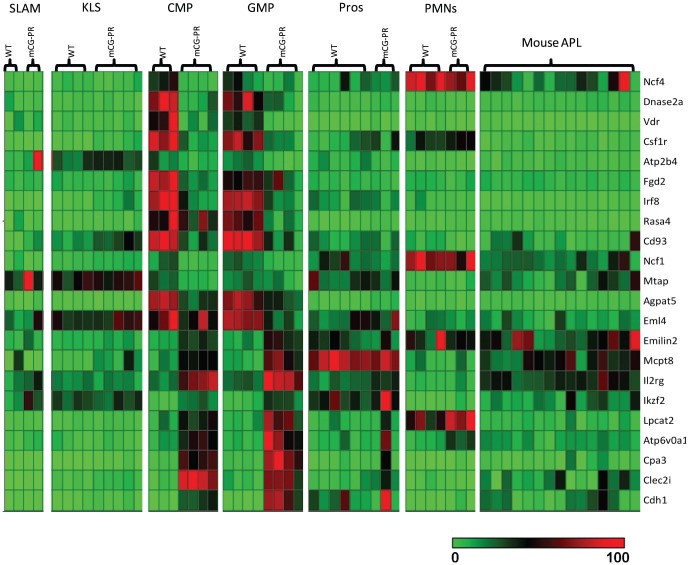
A supervised heatmap of the 22 genes significantly dysregulated in both the CMP and GMP compartments. We created a supervised clustering using SPOTFIRE of the 22 genes that were significantly dysregulated (unadjusted p value<0.001, fold change ≥2) in both the CMP and GMP ANOVA results comparing WT vs. *Ctsg-PML-RARA* samples. The supervised heatmap was created by plotting the relative expression of each of these genes to each other as a relative percentage, rather than z-score averaging, since the differences in expression levels between some genes was large, and z-score averaging inappropriately highlighted the genes with the highest expression levels. The legend is shown below the heatmap, with minimally expressed genes in green, and highly expressed genes in red.

To highlight the changes in gene expression in the CMP and GMP populations, we performed a supervised clustering analysis of the twenty-two genes that were significantly dysregulated (unadjusted p value<0.001, fold change ≥2) in both the CMP and GMP ANOVA results (the supervised heatmap was created by plotting the relative expression of each of these genes to each other) (Figure 4). Interestingly, this gene expression signature is not present in earlier cell populations (KLS or SLAM), in more mature myeloid cells (promyelocytes and neutrophils), nor the leukemic cells from the *Ctsg-PML-RARA* mice. Also, there is no concordant overlap between genes dysregulated in the CMP or GMP compartments versus promyelocytes.

To further validate the changes in gene expression in the CMP and GMP compartments, we performed supervised clustering analyses of all the significantly dysregulated genes by ANOVA (unadjusted p value<0.001, fold change ≥2) from the GMP and CMP compartments individually (these heatmaps were created by traditional z-score scaling). As expected, the clustering with genes dysregulated in either the CMP or GMP compartment segregated both the GMP and CMP populations, but not the MEP populations, by genotype (Figure S7A and S7B). Thus, the expression of *PML-RARA* in *Ctsg-PML-RARA* mice has clear transcriptional consequences that occur early in myeloid development and are specific for the CMP and GMP cell populations. These transcriptional changes, in turn, may influence the gene expression profile of more mature myeloid cells, just as gene expression changes driven by the presence of *PML-RARA* expression in earlier hematopoietic precursors may shape the distinct expression signature seen in the CMP and GMP compartments.

**Figure 5:**
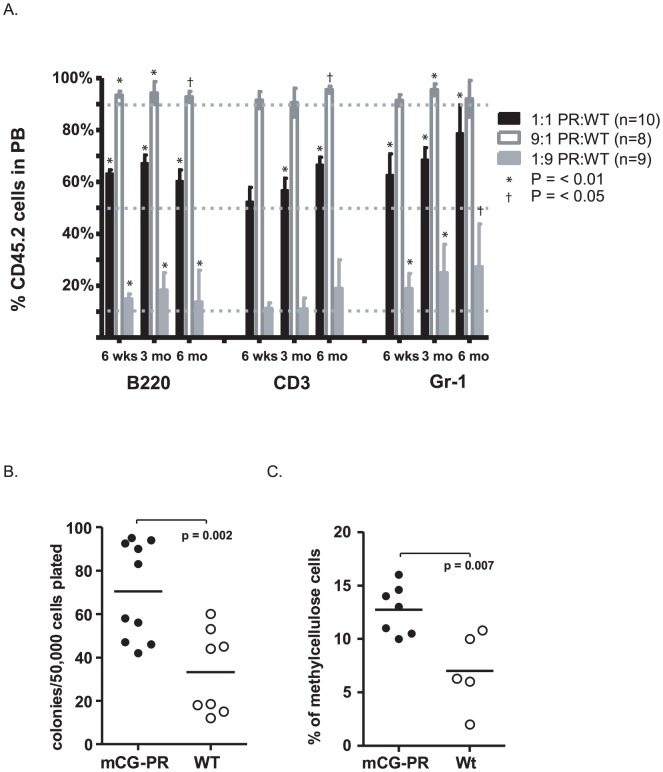
Effects of *Ctsg-PML-RARA* on multi-lineage hematopoiesis. A. Effects on myeloid and lymphoid lineage hematopoiesis. Bone marrow cells from indicated mice at 6 weeks of age were mixed at ratios of 1∶1, 9∶1, or 1∶9 with competitor CD45.1^+^/CD45.2^+^ bone marrow cells from sex- and age-matched mice. These cells were transplanted into sex-matched, 6-week-old, lethally irradiated CD45.1^+^ recipients. At the indicated time points, peripheral blood was assessed for ratios of CD45.2^+^ and CD45.1^+^/CD45.2^+^ white blood cells within the B220^+^, CD3^+^, or Gr1^+^ compartments. One sample, two-tailed t-test compared outcomes with the expected values of 10%, 50%, or 90%. Alpha was set at 0.05. Time points with p<0.01 (*) and p<0.05 (†) are indicated. B–C. Effect of *Ctsg-PML-RARA* on erythroid lineage hematopoiesis. Bone marrow cells from 6-week-old and 8-week-old, healthy mice were plated in methylcellulose containing erythropoietin. B. Total colonies after one week in culture (each data point represents results from an individual mouse in three combined experiments). Results of paired t-test between WT and mCG-PR samples are shown (p-value = 0.002). C. After one week in culture, total colony cells were washed and the number of immature erythrocytes (cKit^+^CD71^dim^) was assessed. Results of a paired t-test between WT and mCG-PR samples are shown (p-value = 0.007).

Secondly, we assessed the function of *Ctsg-PML-RARA* bone marrow cells after competitive transplantation. We transplanted total bone marrow from healthy 6-week-old (i.e. non-leukemic) *Ctsg-PML-RARA* mice (CD45.2^+^) at ratios of 1∶9, 1∶1, and 9∶1 with competitor Ly5.1/Ly5.2 bone marrow (CD45.1^+^/CD45.2^+^) into Ly5.1 recipients (CD45.1^+^). Peripheral blood was assessed at 6 weeks, 3 months, and 6 months post-transplant. Four mice developed leukemia between 3 and 6 months, and could not be analyzed further. As expected, we noted a consistent expansion of *Ctsg-PML-RARA* ^+^ cells within the Ly6g^+^ (myeloid) compartment at all time points tested (Figure 5A). However, we also observed an expansion of *Ctsg-PML-RARA* ^+^ cells within the CD3^+^ and B220^+^ compartments at 3 and 6 months (Figure 5A).

Thirdly, we examined the function of erythroid progenitors derived from *Ctsg-PML-RARA* mice. Total *Ctsg-PML-RARA* bone marrow cells contained significantly more BFU-Es than wild-type littermate controls (Figure 5B). There was no difference in the percentage of mature erythroid cells (Ter119^+^CD71^high^, 24%±8% vs 37%±18%, p = 0.1; Figure S8A) in these colonies, although *Ctsg-PML-RARA* BFU-E colonies contained more immature erythroid cells (cKit^+^CD71^dim^, 12.7%±2.3% vs 7%±3.5%, p = 0.007; Figure 5C) and more myelomonocytic/monocytic cells (CD11b^+^, 32%±4.4% vs 21%±9.6%, p = 0.02; Figure S8B). The lack of transcriptional changes in flow sorted MEP cells (Tables S5 and S12) may be accounted for by differences between the immunophenotypically defined MEPs and the physiologically defined colony forming cell (which may be less mature than MEP cells). Evaluation of a large cohort of mice (n = 19 *Ctsg-PML-RARA* and 15 wild-type littermate controls) revealed normal hemoglobin levels and red cell size in all mice (Figure S8C and S8D), suggesting that *PML-RARA*-dependent effects on BFU-Es are not associated with a loss of erythroid homeostasis *in vivo*. These results parallel our previous finding that myeloid CFUs are increased in healthy pre-leukemic *Ctsg-PML-RARA* mice, but do not lead directly to increased numbers of circulating neutrophils [23], [24].

**Figure 6:**
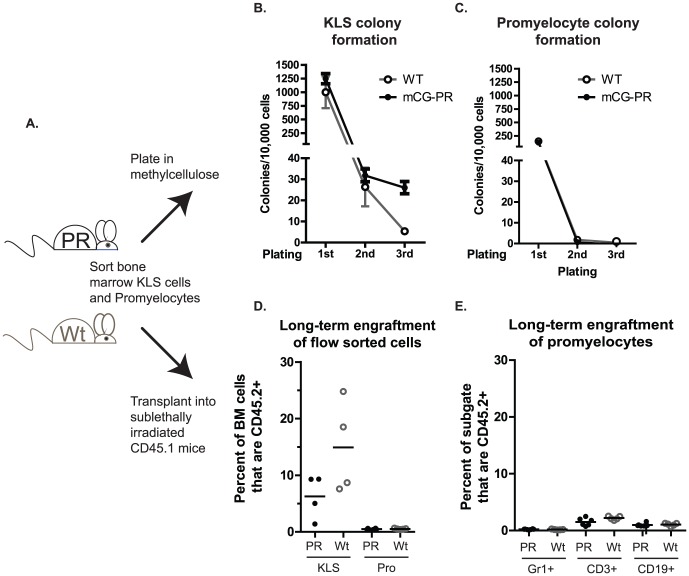
Effects of *Ctsg-PML-RARA* on flow-sorted promyelocytes. A. Experimental schema. Bone marrow cells from *Ctsg-PML-RARA* and littermate WT controls were harvested and KLS and promyelocytes were purified by flow sorting. Aliquots from individual donor mice were plated in methylcellulose containing myeloid cytokines (SCF, IL-3, IL-6, Epo). KLS cells were combined from all donors by genotype and 3,000 cells/recipient were transferred to sub-lethally irradiated Ly5.1 mice (4 recipients per genotype). Purified promyelocytes were separated into two pools, and 50,000 cells/recipient were transferred into sub-lethally irradiated Ly5.1 mice. B. Total CFUs per 10,000 KLS cells plated by week. Every 7 days, total colonies were counted. Total cells were then collected in warm (37°) media and serially replated. C. Total CFUs per 10,000 promyelocytes plated by week. D. Percentage of donor CD45.2^+^ cells in total bone marrow cells of individual recipient mice 12 weeks after engraftment, with indicated flow sorted cells. All mice that received promyelocytes had <1% CD45.2^+^ cells. E. Percentage of CD45.2^+^ cells within the Gr1^+^, CD3^+^, and CD19^+^ bone marrow cells of mice engrafted with promyelocytes.

Finally, we asked whether promyelocyte self-renewal was altered in *Ctsg-PML-RARA* mice. As expected, we observed that KLS cells from *Ctsg-PML-RARA* mice contained increased numbers of CFUs compared with purified promyelocytes (Figure 6B and 6C). We also observed a trend toward increased numbers of CFUs in *Ctsg-PML-RARA* KLS cells compared with wild-type KLS cells. Serial re-plating was detected with *Ctsg-PML-RARA* KLS cells, as expected, but was not found with *Ctsg-PML-RARA* promyelocytes (Figure 6C). Transplantation of pools of donor KLS cells (3,000 cells per sub-lethally irradiated Ly5.1 recipient) from either WT or *Ctsg-PML-RARA* mice led to multi-lineage engraftment, as expected (Figure 6D and Figure S9A–C). In contrast, transplantation of 50,000 purified promyelocytes led to trace engraftment (<1% of bone marrow leukocytes) regardless of genotype, which was not myeloid restricted (Figure 6D–E and Figure S9D–F).

## Discussion

The fusion protein *PML-RARA* is associated exclusively with myeloid leukemia in both humans and mice (unlike *BCR-ABL* and *MLL* fusion proteins, which can lead to myeloid or lymphoid leukemias) [2], [3], [5], [25], [26], [27]. There has therefore been much speculation regarding whether the leukemic cell of origin is a committed progenitor (e.g. a promyelocyte) or multipotent progenitor [5], [6], [7], [8], [10], [14], [28]. The data presented here show that when *PML-RARA* is inserted into the murine *Ctsg* locus, it begins to be expressed in KLS cells, where it alters myeloid, lymphoid, and erythroid hematopoiesis (biologically confirming its activity in multipotent cells). It is massively upregulated in CMP and GMP cells, where it significantly alters the expression of a number of genes. In contrast, *PML-RARA* expression in promyelocytes is not associated with a striking change in gene expression or inappropriate self-renewal. The studies described here define the hematopoietic compartments that are initially exposed to (and perturbed by) *PML-RARA* in healthy, pre-leukemic *Ctsg-PML-RARA* mice; the immunophenotype and cytomorphology of leukemia initiating cells within the subsequent leukemias have been defined elsewhere, and are not addressed in this analysis [8], [9].

One of the important arguments used to support a committed progenitor as the leukemic cell of origin has been the successful generation of leukemia using *CTSG*, *Ctsg*, and *S100A8* loci, which were thought to target *PML-RARA* to the promyelocyte compartment [10]. However, we and others have now found that in *Ctsg-PML-RARA* mice, *PML-RARA* is consistently expressed in cells within the KLS compartment, [8], and is strikingly upregulated in committed myeloid progenitor cells. We have therefore reevaluated the expression patterns of the genes used to direct *PML-RARA* expression in mice: *PML-RARA* directed from the *CTSG*, *Ctsg* and *S100A8* (MRP8) loci cause APL; expression directed by regulatory elements from the *cFes* and *Itgam* (CD11b) loci do not [1], [2], [3], [19], [20]. *Ctsg*, *CTSG*, *S100a8*, and *S100A8* are all expressed in multipotent progenitor compartments, while *cFes* and *Itgam* display low to absent levels of expression in KLS cells, with maximal expression in neutrophils. This suggests that APL development may require *PML-RARA* expression in an early hematopoietic compartment, and that induction of expression at the promyelocyte stage is insufficient to initiate leukemia, perhaps because PML-RARA cannot reverse the commitment of terminally differentiated neutrophils to die.

Other authors have evaluated the cell-specific effects of *PML-RARA* and MLL fusion transcripts using viral transduction [4], [29], [30], [31]. We have focused our analysis on transgenic mouse models because interpretation of viral-transduction models is complicated both by the heterogeneity of the bone marrow cells transduced, and by the toxicity of *PML-RARA*, which is variable among cell types [28], [32], [33].

Importantly, this work addresses a different question than the work of Guibal et al., which defined the immunophenotype of a committed myeloid progenitor (cKit^+^CD34^+^FcγRII/III^+^Ly6g^int^) as the leukemia-initiating cell in the h*MRP8-PML-RARA* transgenic mouse model of APL. Here we show the transcriptional and functional consequences of *PML-RARA* expression in *Ctsg-PML-RARA* pre-leukemic cells. These findings are not mutually exclusive, since the hematopoietic compartment susceptible to *PML-RARA* transformation may not be at the same developmental stage as the resultant leukemia [9].

Our work clarifies and expands the findings of Wojiski et al. [8] Using semi-quantitative RT-PCR, they observed *PML-RARA* expression in the KLS cells of *Ctsg-PML-RARA* mice. Their functional studies revealed increased myeloid self-renewal in flow-sorted KLS, CMP, and GMP cells from *Ctsg-PML-RARA* mice. They used a PCR-based protocol to demonstrate long-term engraftment of flow-sorted *Ctsg-PML-RARA* promyelocytes (Lin^−^Sca^−^Ly6g^+^cKit^+^CD34^+^ cells) in sub-lethally irradiated recipient B6 mice. However, this strategy does not determine whether the engrafted cells are myeloid-restricted (i.e. derived from promyelocytes) or whether they are multi-lineage (i.e. arising from stem/progenitor cells that contaminated the purified “promyelocytes"). In contrast, we used Ly5.1 mice as the recipients for our purified promyelocyte populations, and found that even large numbers of promyelocytes (50,000 purified cells per recipient) were insufficient to lead to long-term engraftment of myeloid restricted cells. Our data therefore do not support the idea that the promyelocytes from *Ctsg-PML-RARA* mice have altered self-renewal properties that contribute to the expansion of these cells *in vivo*.

There are no standard immunophenotypic markers for defining murine promyelocytes, and our flow-sorting strategy for promyelocytes was different than that used by Wojiski et al.; however, we extensively validated our approach using both expression data from genes known to be developmentally-regulated during myeloid development as well as traditional cytomorphology, in which a trained hematopathologist performed blinded differentials (Figure S1) [8], [34]. Using these techniques, we demonstrate that we have greatly enriched murine promyelocytes using our flow-sorting strategy; however, it remains possible that the different strategy used by Wojiski et al. enriches for an earlier myeloid population that could account for the different results regarding “promyelocyte" self-renewal between our studies.

Nonetheless, our data demonstrate that in healthy, pre-leukemic *Ctsg-PML-RARA* mice, *PML-RARA* is first expressed in very early hematopoietic stem/[progenitor cells (KLS cells), and that its expression continues throughout myeloid development (CMPs, GMPs, promyelocytes, and neutrophils). Biological effects of *PML-RARA* can be seen in pre-leukemic KLS cells, CMPs and GMPs. Thus, in this model system, it is highly likely that PML-RARA initiates leukemia in very early hematopoietic cells, and not in promyelocytes, as originally predicted. Although we cannot formally exclude the possibility that pre-leukemic promyelocytes acquire self-renewal properties, it seems very unlikely, based on recent data that some human APL cells have TCR rearrangements, and express T lineage genes [14]. We suggest that the *Ctsg-PML-RARA* mouse model may recapitulate the physiologic timing of *PML-RARA* activation in very primitive human HPSCs that are capable of giving rise to both lymphoid and myeloid cells.

**Figure 7:**
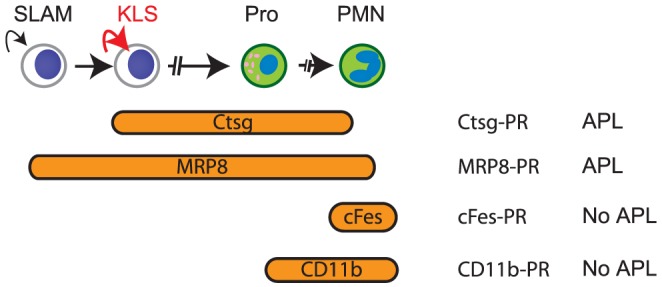
Model of APL development in *Ctsg-PML-RARA* mice. Murine APL requires the expression of *PML-RARA* in a multipotent progenitor, and cooperation of *PML-RARA* with myeloid restricted elements; expression of *PML-RARA* within promyelocytes is insufficient to cause leukemia. Orange ellipses indicate compartments with expression of the indicated genes used to direct expression in transgenic models. Red arrows indicate inappropriate self-renewal resulting from *Ctsg-PML-RARA* expression. Broken arrows indicate that multiple differentiation steps exist between indicated compartments. SLAM: lineage negative/Kit^+^/Sca-1^+^/CD150^+^/CD41^−^/CD48^−^. KLS: c-Kit^+^/lineage negative/Sca-1^+^. Pro: promyelocyte. PMN: polymorphic neutrophil. Concept after Lane and Ley [28].

*PML-RARA* does not appear to alter normal hematopoietic homeostatic feedback or cause increasing cell *numbers* within an immunophenotypically-defined multipotent progenitor compartment (although healthy *Ctsg-PML-RARA* mice do have a subtle increase in bone marrow promyelocytes) [2], [8], [11], [24]. Neither we, nor others, have observed increased numbers of KLS, GMP, CMP or MEP cells in mouse models of APL [8], [20], [24], [35]. However, we have identified a competitive advantage in multiple hematopoietic lineages following transplantation [11], and increased numbers of myeloid [26], [36] and erythroid BFUs in the marrow of *Ctsg-PML-RARA* mice. Since *Ctsg-PML-RARA* mice virtually never develop lymphocytic leukemia or erythroleukemia, our data suggests that the myeloid restriction of leukemia must be explained by myeloid-restricted genes or proteins that cooperate with PML-RARA in leukemic cells (Figure 7). Although neutrophil elastase is known to be one of the critical interacting proteins, further investigation will be needed to fully define all of the cooperating elements [23], [28].

## Methods

### Mice

Mice expressing *PML-RARA* from the murine *Ctsg* locus (*Ctsg-PML-RARA*) and *Ctsg* deficient mice (CG-KO) have been previously reported [2], [37]. The *Ctsg-PML-RARA* transgenic mice used in this study were backcrossed more than 12 generations into the C57Bl/6 Taconic background [38]. The Washington University Animal Studies Committee approved all animal experiments.

### Flow Sorting, Expression Array Profiling, and RT-PCR Validation

Bone marrow cells from individual mice were harvested from both femurs and tibia of 6-week-old and 13-week-old mice and prepared previously described [11], [24]. Standard red blood cell lysis was performed with ACK lysis buffer (0.15 M NH_4_Cl, 10 mM KHC0_3_, 0.1 mM Na_2_EDTA) on ice for 10 minutes. Non-specific staining was blocked with Miltenyi FcR Blocking Reagent for mouse (Auburn, CA). Isotype-matched antibodies were used as negative controls and the Fluorescence Minus One (FMO) strategy was used to set appropriate gates. Flow sorting was performed on a Reflection high-speed cell sorter (i-Cyt, Champaign, IL). Cells were sorted directly into Trizol (Invitrogen, Carlsbad, CA). Cells were stained by standard protocols with the following antibodies (eBioscience unless otherwise noted): KLS (c-Kit^+^/lineage negative/Sca-1^+^) cells were assessed using the following lineage markers: FITC-conjugated αGr-1, αTer119, αCD3, αCD4, αCD8, αB220, αCD19, and αCD127; APC-αc-Kit, and PE-αSca-1. CMPs (Lin^−^Sca-1^−^cKit^+^CD34^+^FcγRII/III^lo^), GMPs (Lin^−^Sca-1^−^cKit^+^CD34^+^FcγRII/III^hi^), megakaryocyte-erythrocyte progenitors (MEPs; Lin^−^Sca-1^−^cKit^+^CD34^−^FcγRII/III^lo^) were done with the same antibody cocktail as KLS with addition of APC-conjugated αFcγRII/III (clone 93). SLAM (CD150^+^/CD41^−^/CD48^−^ KLS) cells were assessed using KLS staining as above, except for PerCP-Cy5.5–conjugated αSca-1, with the addition of FITC-αCD41, FITC-αCD48 and PE-αCD150 antibodies. Promyelocyte (Gr1^int^SCC^int^B220^−^CD115^−^Ter119^−^) and neutrophil (Gr1^+^SCC^high^B220^−^CD115^−^Ter119^−^) sorting was done using APC-αGr-1, PE-αCD115, FITC-αB220 and PE-Cy7-αTer119. Additional cells from both the promyelocyte and neutrophils fraction were sorted into FACS buffer for morphological validation of cytospins. Samples analyzed in expression array profiling were generated during five separate flow-sorting experiments to limit technical bias. 15 mouse *Ctsg-PML-RARA* leukemia samples were prepared as previously described [38].

For expression array profiling, total cellular RNA was purified using TRIzol reagent (Invitrogen), quantified using UV spectroscopy (Nanodrop Technologies), and qualitatively assessed using an Experion Bioanalyzer. Amplified cDNA was prepared from 20 ng total RNA using the whole transcript WT-Ovation RNA Amplification System and biotin-labeled using the Encore Biotin Module, both from NuGen Technologies, according to the manufacturer's instructions. Labeled targets were then hybridized to Mouse Exon 1.0 ST arrays (Affymetrix), washed, stained, and scanned using standard protocols from the Siteman Cancer Center, Molecular and Genomic Analysis Core Facility (http://pathology.wustl.edu/research/cores/lcg/index.php). Affymetrix Expression Console software was used to process array images, export signal data, and evaluate image and data quality relative to standard Affymetrix quality control metrics. Exon array data for all samples used in this study have been deposited on GEO (http://www.ncbi.nlm.nih.gov/geo/; SuperSeries accession numbers GSE26131 and GSE40022).

For *Ctsg* qRT-PCR, we performed standard, two-step RT-PCR, including the Genomic DNA Wipeout step, according to the manufacturer's instructions using *Ctsg* QuantiTect Primers in conjunction with the QuantiTect SYBER Green RT-PCR Kit (both from Qiagen, Germantown, Maryland). For PML-RARA qRT-PCR, we also used the Qiagen RT-PCR kit with the following primers: Forward TCTTCCTGCCCAACAGCAA, Reverse GCTTGTAGATGCGGGGTAGAG. We used GAPDH as the PCR control with the following primers: Forward TGCACCACCAACTGCTTAG, Reverse GGATGCAGGGATGATGTTC.

### Bone marrow Transplantation

Cells used for competitive repopulation studies were injected retroorbitally, as previously described [11], [24]. Two separate experiments were performed, and the data was combined for analysis.

Determination of engraftment of flow sorted bone marrow cells is described in Figure S9. Recipient Ly5.1 mice received either lethal (1,100 cGy) or sub-lethal (350 cGy) irradiation 24 hours prior to transplantation with either 1×10^6^ total bone marrow cells, 3,000 KLS cells or 50,000 promyelocytes/mouse. At least 20,000 events (peripheral blood) and 40,000 events (bone marrow) per recipient mouse were collected for analysis.

### Hematopoietic Progenitor Assays

Bone marrow cells from 6-week-old and 8-week-old, healthy *Ctsg-PML-RARA* mice and littermate controls were collected and plated (in duplicate) in 1.1 ml of methylcellulose medium at 41.5×10^3^ cells/ml (MethoCult M3334, StemCell Technologies, Vancouver, Canada) or 8.3×10^3^/ml (MethoCult 3434) [37]. Colonies with >30 cells were counted on day 7. Colonies were collected in 37° DMEM and assessed by flow cytometry using the following antibodies and FACS Scan (Beckman-Dickenson, Franklin Lakes, NJ): FITC-αTer119 (BD Pharmingen, Franklin Lakes, NJ, Ly-76), PE-αCD71 (eBioscience, R17217), PE-αCD11b (eBioscience, M1/70), and APC-αcKit (eBioscience, 2B8), or serially replated.

### Statistical analysis

Expression array analysis: exon-level summary was generated using the RMA algorithm in the Affymetrix Expression Console (Affymetrix Inc., USA). Only core probesets were used in order to limit the analysis within well-annotated exons with the exception of *Ly6g*, whose 2 probesets are within the extended probesets group. Probesets having an expression signal less than 200 in all samples were removed and an unsupervised hierarchical clustering analysis was performed using z-score normalized expression values in SPOTFIRE (Decision Site version 9.1.1, Somerville, USA) or with the Partek Genomics Suite (Saint Louis, MO) with the expression of each gene standardized to a mean of 0 and standard deviation of 1 and using the Pearson dissimilarity as a distance measure. Results did not segregate based on age of the mice used in this analysis (6-week-old vs 13-week-old), and so the data were combined. Supervised clustering analyses were done using SPOTFIRE as described in the text. Comparison of cell populations using ANOVA was done with Partek with fold change ≥2 and unadjusted p-values as noted in the text. Comparisons of cell populations using SAM Version 4.0 (http://www-stat.stanford.edu/~tibs/SAM/, Palo Alto, CA) as an Excel add-in performing two-class unpaired analyses with unlogged data, 100 permutations, standard regression and fold change ≥2 with false discovery rates and q-values noted in the text. Competitive repopulation analysis: a one sample, two-tailed t-test with alpha set at 0.05 compared outcomes with expected values of 10%, 50%, and 90% (Prism, Graphpad 5, La Jolla, CA). CFU-E analysis was done with a paired t-test (Excel, Microsoft, Seattle, WA).

## Supporting Information

Figure S1 **Murine promyelocyte and neutrophil flow sorting strategy.** A. Total bone marrow cells were labeled with B220, Ter119, CD115 and Gr1 and early myeloid cells/promyelocytes (Pros) and neutrophils (PMNs) were identified as B220^−^, Ter119^−^, CD115^−^ cells that are either BSC^int^Gr1^int^ (Pros), or BSC^high^Gr1^high^ (PMNs). B. Results of 200 cell differential counts by a blinded hematopathologist from 4 Pros (2 WT and 2 *Ctsg-PML-RARA*) and 5 PMNs (3 WT and 2 *Ctsg-PML-RARA*) separate sorted samples. C. Representative cytomorphology of WT and *Ctsg-PML-RARA* (labeled PR) Pros samples counted in B (1,000×). D. Representative cytomorphology of WT and *Ctsg-PML-RARA* (labeled PR) PMNs samples counted in B (1,000×).
(EPS)

Figure S2 **Validation of** ***Ctsg*** **and** ***PML-RARA*** **expression using quantitative reverse-transcriptase PCR.** A. *Ctsg* expression was validated with quantitative RT-PCR normalized to *Gapdh*. B. Quantitative RT-PCR using *PML-RARA* specific primers normalized to *Gapdh* in the indicated mice and Nugen amplified mRNA. C. Agarose gel of PCR products from Panel B. *PML-RARA* expected size is 145 bp. Markers are 100 and 200 base-pairs.
(EPS)

Figure S3 **Expression of** ***Ly6g*** **,** ***Kit*** **,** ***Flt3*** **and** ***Cd34*** **in flow-sorted bone marrow cells and mouse leukemia samples.** Expression profile in indicated WT and *Ctsg-PML-RARA* (labeled mCG-PR) flow-sorted bone marrow cells and 15 *Ctsg-PML-RARA* leukemia samples (labeled Mouse APL) using Nugen amplified mRNA and Affymetrix Mouse Exon 1.0ST arrays. We plotted *Ly6g*, *Kit*, *Flt3* and *Cd34* expression using representative probesets.
(EPS)

Figure S4 **Expression of 7 developmentally-regulated myeloid genes in flow-sorted bone marrow cells.** Expression profile in indicated WT and *Ctsg-PML-RARA* (labeled mCG-PR) flow-sorted bone marrow cells and 15 *Ctsg-PML-RARA* leukemia samples (labeled Mouse APL) using Nugen amplified mRNA and Affymetrix Mouse Exon 1.0ST arrays. We plotted *Elane*, *Prtn3*, *Mpo*, *Ltf*, *Mmp9*, *Lyz2*, and *Fpr1* expression using representative probesets.
(EPS)

Figure S5 **Expression profile of flow-sorted** ***Ctsg-PML-RARA*** **and WT bone marrow cells by unsupervised clustering analyses.** Bone marrow cells were flow sorted as described in Figure 1, and analyzed using Affymetrix Exon 1.0ST arrays. A: Unsupervised hierarchal clustering using SPOTFIRE of expression array data from flow-sorted SLAM and KLS cells from littermate 6-week-old and 13-week-old healthy WT (−/−) vs. mCG-PR mice (+/−). Clustering occurs by genotype within the KLS samples, but not the SLAM samples. Samples were generated during two independent flow-sorting experiments to minimize technical bias. B. Unsupervised hierarchal clustering using SPOTFIRE of expression array data of flow-sorted promyelocytes cells from littermate 8-week-old healthy WT (−/−) vs. mCG-PR mice (+/−). Clusters do not segregate by genotype. Samples were generated with three independent flow-sorting experiments to minimize technical bias. The legend is shown below the heatmaps with downregulated genes in green and upregulated genes in red.
(EPS)

Figure S6 **Unsupervised heatmap clustering analyses for WT vs. Ctsg-PML-RARA CMP, GMP and MEP samples.** Bone marrow cells were flow sorted as described in the Methods section, and analyzed using Affymetrix Exon 1.0ST arrays. An unsupervised clustering analysis was performed using Partek with Pearson dissimilarity as the distance measure. Probesets with signal intensity of less than 200 in all samples were removed from the analyses. The legend is shown below each heatmap with downregulated genes in green and upregulated genes in red. A. Unsupervised heatmap clustering analyses for CMP WT vs. *Ctsg-PML-RARA* samples. B. Unsupervised heatmap clustering analyses for GMP WT vs. *Ctsg-PML-RARA* samples. C. Unsupervised heatmap clustering analyses for MEP WT vs. *Ctsg-PML-RARA* samples.
(EPS)

Figure S7 **Individual supervised clustering analyses for the significantly dysregulated genes by ANOVA from the GMP and CMP comparisons between WT and Ctsg-PML-RARA samples.** These heatmaps were created by traditional z-score scaling using SPOTFIRE. The clustering with genes dysregulated in either the CMP or GMP compartment by ANOVA (with unadjusted p value<0.001, fold change ≥2) segregated both the GMP and CMP populations, but not the MEP populations, by genotype. The legend is shown below the heatmaps with downregulated genes in green and upregulated genes in red. A. Supervised clustering heatmap using genes dysregulated by ANOVA comparison between WT and *Ctsg-PML-RARA* CMP populations. B. Supervised clustering heatmap using genes dysregulated by ANOVA comparison between WT and *Ctsg-PML-RARA* GMP populations.
(EPS)

Figure S8 **Erythroid colony formation (CFU-E) in** ***Ctsg-PML-RARA*** **vs. WT mice.** Bone marrow cells from 6-week-old and 8-week-old, healthy mice were plated in methylcellulose containing erythropoietin (Methocult 3334). Results of paired t-tests are shown in each panel. A. Immunophenotypes of cells in these colonies were assessed as indicated: A. Ter119^+^CD71^high^ (mature erythrocytes) and B. CD11b^+^ (myelomonocytic/monocytic cells). C. and D. Hemoglobin and mean corpuscular volume (MCV) values were determined in a cohort of healthy 4-month-old mice with the indicated genotypes.
(EPS)

Figure S9 **Examples of bone marrow immunophenotypes following transplantation of flow sorted KLS and promyelocytes.** Bone marrow KLS and promyelocytes were sorted from littermate *Ctsg-PML-RARA* (mCG-PR) and WT mice and transplanted into sub-lethally irradiated Ly5.1 recipients (Figure 6). Twelve weeks after transplantation, bone marrow cells were harvested and assessed for Gr1, CD3, CD19, CD45.2 and CD45.1 expression. At least 40,000 events were collected per recipient. A–C. Representative recipient of KLS transplantation. D–F. Representative recipient of promyelocyte transplantation.
(EPS)

Data S1 **Comparative gene expression analysis between cell populations derived from** ***Ctsg-PML-RARA*** **versus WT mice.** The supplementary data includes a summary of gene expression differences (by ANOVA and SAM analysis) for comparisons between *Ctsg-PML-RARA* and WT SLAM, KLS, CMP, GMP, MEP and promyelocyte cell populations. The analysis includes a comparison of dysregulated genes to previously identified PML-RARA binding sites and to known dysregulated mRNA abundance identified in primary human APL samples [39], [40].
(DOCX)

Table S1 ***Ctsg-PML-RARA*** **versus WT SLAM ANOVA Results.**
(XLSX)

Table S2 ***Ctsg-PML-RARA*** **versus WT KLS ANOVA Results.**
(XLSX)

Table S3 ***Ctsg-PML-RARA*** **versus WT CMP ANOVA Results.**
(XLSX)

Table S4 ***Ctsg-PML-RARA*** **versus WT GMP ANOVA Results.**
(XLSX)

Table S5 ***Ctsg-PML-RARA*** **versus WT MEP ANOVA Results.**
(XLSX)

Table S6 ***Ctsg-PML-RARA*** **versus WT Promyelocyte ANOVA Results.**
(XLSX)

Table S7 **Concordant Dysregulated Genes in both the CMP and GMP Compartments by ANOVA.**
(XLSX)

Table S8 ***Ctsg-PML-RARA*** **versus WT SLAM SAM Results.**
(XLSX)

Table S9 ***Ctsg-PML-RARA*** **versus WT KLS SAM Results.**
(XLSX)

Table S10 ***Ctsg-PML-RARA*** **versus WT CMP SAM Results.**
(XLSX)

Table S11 ***Ctsg-PML-RARA*** **versus WT GMP SAM Results.**
(XLSX)

Table S12 ***Ctsg-PML-RARA*** **versus WT MEP SAM Results.**
(XLSX)

Table S13 ***Ctsg-PML-RARA*** **versus WT Promyelocyte SAM Results.**
(XLSX)

## References

[1] Grisolano JL, Wesselschmidt RL, Pelicci PG, Ley TJ (1997) Altered myeloid development and acute leukemia in transgenic mice expressing PML-RAR alpha under control of cathepsin G regulatory sequences. Blood 89: 376–387. 9002938

[2] Westervelt P, Lane AA, Pollock JL, Oldfather K, Holt MS, et al (2003) High-penetrance mouse model of acute promyelocytic leukemia with very low levels of PML-RARalpha expression. Blood 102: 1857–1865. 12750176 10.1182/blood-2002-12-3779

[3] Brown D, Kogan S, Lagasse E, Weissman I, Alcalay M, et al (1997) A PMLRARalpha transgene initiates murine acute promyelocytic leukemia. Proc Natl Acad Sci U S A 94: 2551–2556. 9122233 10.1073/pnas.94.6.2551 PMC20126

[4] Du C, Redner RL, Cooke MP, Lavau C (1999) Overexpression of wild-type retinoic acid receptor alpha (RARalpha) recapitulates retinoic acid-sensitive transformation of primary myeloid progenitors by acute promyelocytic leukemia RARalpha-fusion genes. Blood 94: 793–802. 10397747

[5] Kogan SC (2007) Mouse models of acute promyelocytic leukemia. Curr Top Microbiol Immunol 313: 3–29. 17217036 10.1007/978-3-540-34594-7_2

[6] Westervelt P, Ley TJ (1999) Seed versus soil: the importance of the target cell for transgenic models of human leukemias. Blood 93: 2143–2148. 10090920

[7] Turhan A, Lemoine F, Debert C, Bonnet M, Baillou C, et al (1995) Highly purified primitive hematopoietic stem cells are PML-RARA negative and generate nonclonal progenitors in acute promyelocytic leukemia. Blood 85: 2154–2161. 7536493

[8] Wojiski S, Guibal FC, Kindler T, Lee BH, Jesneck JL, et al (2009) PML-RARalpha initiates leukemia by conferring properties of self-renewal to committed promyelocytic progenitors. Leukemia 23: 1462–1471. 19322209 10.1038/leu.2009.63 PMC2914549

[9] Guibal FC, Alberich-Jorda M, Hirai H, Ebralidze A, Levantini E, et al (2009) Identification of a myeloid committed progenitor as the cancer-initiating cell in acute promyelocytic leukemia. Blood 114: 5415–5425. 19797526 10.1182/blood-2008-10-182071 PMC2798860

[10] Grimwade D, Enver T (2004) Acute promyelocytic leukemia: where does it stem from? Leukemia 18: 375–384. 14737069 10.1038/sj.leu.2403234

[11] Welch JS, Yuan W, Ley TJ (2011) PML-RARA can increase hematopoietic self-renewal without causing a myeloproliferative disease in mice. J Clin Invest 121. 10.1172/JCI42953 PMC3068978 21364283

[12] Ito K, Bernardi R, Morotti A, Matsuoka S, Saglio G, et al (2008) PML targeting eradicates quiescent leukaemia-initiating cells. Nature 453: 1072–1078. 18469801 10.1038/nature07016 PMC2712082

[13] Bonnet D, Dick JE (1997) Human acute myeloid leukemia is organized as a hierarchy that originates from a primitive hematopoietic cell. Nat Med 3: 730–737. 9212098 10.1038/nm0797-730

[14] Chapiro E, Delabesse E, Asnafi V, Millien C, Davi F, et al (2006) Expression of T-lineage-affiliated transcripts and TCR rearrangements in acute promyelocytic leukemia: implications for the cellular target of t(15;17). Blood 108: 3484–3493. 16857994 10.1182/blood-2005-09-009977

[15] Guglielmi C, Martelli MP, Diverio D, Fenu S, Vegna ML, et al (1998) Immunophenotype of adult and childhood acute promyelocytic leukaemia: correlation with morphology, type of PML gene breakpoint and clinical outcome. A cooperative Italian study on 196 cases. Br J Haematol 102: 1035–1041. 9734655 10.1046/j.1365-2141.1998.00871.x

[16] Theilgaard-Monch K, Jacobsen LC, Borup R, Rasmussen T, Bjerregaard MD, et al (2005) The transcriptional program of terminal granulocytic differentiation. Blood 105: 1785–1796. 15514007 10.1182/blood-2004-08-3346

[17] Borregaard N, Cowland JB (1997) Granules of the human neutrophilic polymorphonuclear leukocyte. Blood 89: 3503–3521. 9160655

[18] Yuan W, Payton JE, Holt MS, Link DC, Watson MA, et al (2007) Commonly dysregulated genes in murine APL cells. Blood 109: 961–970. 17008535 10.1182/blood-2006-07-036640 PMC1785140

[19] Pandolfi PP (1997) Transgenic models of acute myeloid leukemias. Hematology 1997: Education Program of the American Society for Hematology. San Diego, CA.

[20] Early E, Moore MA, Kakizuka A, Nason-Burchenal K, Martin P, et al (1996) Transgenic expression of PML/RARalpha impairs myelopoiesis. Proc Natl Acad Sci U S A 93: 7900–7904. 8755574 10.1073/pnas.93.15.7900 PMC38846

[21] Paredes J, Figueiredo J, Albergaria A, Oliveira P, Carvalho J, et al (2012) Epithelial E- and P-cadherins: Role and clinical significance in cancer. Biochimica et Biophysica Acta (BBA) - Reviews on Cancer 1826: 297–311. 22613680 10.1016/j.bbcan.2012.05.002

[22] Bour G, Gaillard E, Bruck N, Lalevee S, Plassat J-L, et al (2005) Cyclin H binding to the RARA activation function (AF)-2 domain directs phosphorylation of the AF-1 domain by cyclin-dependent kinase 7. Proc Natl Acad Sci U S A 102: 16608–16613. 16275922 10.1073/pnas.0505556102 PMC1283805

[23] Uy GL, Lane AA, Welch JS, Grieselhuber NR, Payton JE, et al (2010) A protease-resistant PML-RAR{alpha} has increased leukemogenic potential in a murine model of acute promyelocytic leukemia. Blood 116: 3604–3610. 20647568 10.1182/blood-2008-11-189282 PMC2981479

[24] Welch JS, Klco JM, Varghese N, Nagarajan R, Ley TJ (2011) Rara haploinsufficiency modestly influences the phenotype of acute promyelocytic leukemia in mice. Blood 117: 2460–2468. 21190992 10.1182/blood-2010-08-300087 PMC3317790

[25] Zhou G-b, Li G, Chen S-j, Chen Z (2007) From dissection of disease pathogenesis to elucidation of mechanisms of targeted therapies: leukemia research in the genomic era. Acta Pharmacol Sin 9 1434–1449. 17723177 10.1111/j.1745-7254.2007.00684.x

[26] Ilaria RL Jr (2004) Animal models of chronic myelogenous leukemia. Hematol Oncol Clin North Am 18: 525–543, vii. 15271391 10.1016/j.hoc.2004.03.003

[27] McCormack E, Bruserud O, Gjertsen BT (2008) Review: genetic models of acute myeloid leukaemia. Oncogene 27: 3765–3779. 18264136 10.1038/onc.2008.16

[28] Lane AA, Ley TJ (2005) Neutrophil elastase is important for PML-retinoic acid receptor alpha activities in early myeloid cells. Mol Cell Biol 25: 23–33. 15601827 10.1128/MCB.25.1.23-33.2005 PMC538790

[29] So CW, Karsunky H, Passegue E, Cozzio A, Weissman IL, et al (2003) MLL-GAS7 transforms multipotent hematopoietic progenitors and induces mixed lineage leukemias in mice. Cancer Cell 3: 161–171. 12620410 10.1016/s1535-6108(03)00019-9

[30] Lavau C, Luo RT, Du C, Thirman MJ (2000) Retrovirus-mediated gene transfer of MLL-ELL transforms primary myeloid progenitors and causes acute myeloid leukemias in mice. Proc Natl Acad Sci U S A 97: 10984–10989. 10995463 10.1073/pnas.190167297 PMC27135

[31] Minucci S, Monestiroli S, Giavara S, Ronzoni S, Marchesi F, et al (2002) PML-RAR induces promyelocytic leukemias with high efficiency following retroviral gene transfer into purified murine hematopoietic progenitors. Blood 100: 2989–2995. 12351412 10.1182/blood-2001-11-0089

[32] He LZ, Tribioli C, Rivi R, Peruzzi D, Pelicci PG, et al (1997) Acute leukemia with promyelocytic features in PML/RARalpha transgenic mice. Proc Natl Acad Sci U S A 94: 5302–5307. 9144232 10.1073/pnas.94.10.5302 PMC24673

[33] Sternsdorf T, Phan VT, Maunakea ML, Ocampo CB, Sohal J, et al (2006) Forced retinoic acid receptor alpha homodimers prime mice for APL-like leukemia. Cancer Cell 9: 81–94. 16473276 10.1016/j.ccr.2005.12.030

[34] McGarry MP, Protheroe CA, Lee JJ (2009) Mouse Hematology: A Laboratory Manual. Long Island, NY: Cold Spring Harbor Laboratory Press. 99 p.

[35] Walter MJ, Park JS, Ries RE, Lau SK, McLellan M, et al (2005) Reduced PU.1 expression causes myeloid progenitor expansion and increased leukemia penetrance in mice expressing PML-RARalpha. Proc Natl Acad Sci U S A 102: 12513–12518. 16113082 10.1073/pnas.0504247102 PMC1188022

[36] Rego EM, Wang ZG, Peruzzi D, He LZ, Cordon-Cardo C, et al (2001) Role of promyelocytic leukemia (PML) protein in tumor suppression. J Exp Med 193: 521–529. 11181703 10.1084/jem.193.4.521 PMC2195907

[37] MacIvor DM, Shapiro SD, Pham CT, Belaaouaj A, Abraham SN, et al (1999) Normal neutrophil function in cathepsin G-deficient mice. Blood 94: 4282–4293. 10590073

[38] Wartman LD, Larson DE, Xiang Z, Ding L, Chen K, et al (2011) Conserved progression mutations revealed by sequencing a mouse acute promyelocytic leukemia genome. J Clin Invest 121: 1445–1455. 21436584 10.1172/JCI45284 PMC3069786

[39] Martens JH, Brinkman AB, Simmer F, Francoijs KJ, Nebbioso A, et al (2010) PML-RARalpha/RXR alters the epigenetic landscape in acute promyelocytic leukemia. Cancer Cell 17: 173–185. 20159609 10.1016/j.ccr.2009.12.042

[40] Payton JE, Grieselhuber NR, Chang LW, Murakami M, Geiss GK, et al (2009) High throughput digital quantification of mRNA abundance in primary human acute myeloid leukemia samples. J Clin Invest 119: 1714–1726. 19451695 10.1172/JCI38248 PMC2689138

