# Supplementary material for: Expression and Function of PML-RARA in the Hematopoietic Progenitor Cells of Ctsg-PML-RARA Mice
Source: PLoS One. 2012 Oct 8;7(10):e46529. doi: 10.1371/journal.pone.0046529 (PMC3466302; doi:10.1371/journal.pone.0046529)
Supplement: Data S1 — Comparative gene expression analysis between cell populations derived from Ctsg-PML-RARA versus WT mice. The supplementary data includes a summary of gene expression differences (by ANOVA and SAM analysis) for comparisons between Ctsg-PML-RARA and WT SLAM, KLS, CMP, GMP, MEP and promyelocyte cell populations. The analysis includes a comparison of dysregulated genes to previously identified PML-RARA binding sites and to known dysregulated mRNA abundance identified in primary human APL samples [39], [40]. (DOCX) [file pone.0046529.s010.docx]

**Data S1. Supplementary Data**

To determine the earliest changes in gene expression that may be driven by *PML-RARA* expression, we compared the gene expression profiles of *Ctsg-PML-RARA* versus WT SLAM cell populations. There were 69 unique, annotated genes that are significantly dysregulated by ANOVA with a p-value < 0.001 and fold change of ≥ 2; however, there were no significantly dysregulated genes by SAM analysis using a fold change of at least 2 and a false discovery rate (FDR) = 0. Of the significantly dysregulated genes by ANOVA, 38/69 are down-regulated (55%) in *Ctsg-PML-RARA* SLAM cells. Only 1/69 KLS ANOVA hits (1%) overlapped with the ChIP binding sites (near 487 unique annotated genes identified in both NB4 cells and 2 human APL samples) identified by Martens et al.[39] This gene is down-regulated in *Ctsg-PML-RARA* mice. However, from our group’s previously published data, only 3 of the top 40 genes (7.5%) dysregulated in primary human APL samples (by mRNA abundance) overlap with the previously identified ChIP binding sites, which demonstrates that although *PML-RARA* acts as a putative repressor of gene expression, its effects on the overall transcriptome are complex.[40]

In the comparison of gene expression between *Ctsg-PML-RARA* and WT KLS cell populations, there were 42 unique, annotated genes that are significantly dysregulated by ANOVA with a p-value < 0.001 and fold change of ≥ 2. Of these genes, 18/42 are down-regulated (43%) in *Ctsg-PML-RARA* mice. Only 2/42 KLS ANOVA hits (5%) overlapped with the previously identified ChIP binding sites.[39] Both of these genes are down-regulated in *Ctsg-PML-RARA* mice. In the SAM analysis (fold change ≥ 2 and a FDR = 0), there were 9 unique genes that were significantly dysregulated in the *Ctsg-PML-RARA* mice, and all are down-regulated in the *Ctsg-PML-RARA* mice.

In the comparison of gene expression between *Ctsg-PML-RARA* and WT CMPs, there were 129 unique, annotated genes that are significantly dysregulated by ANOVA with a p-value < 0.001 and fold change of ≥ 2. Of these genes, 99/129 are down-regulated (77%). Only 7/129 CMP ANOVA hits (5%) overlapped with the previously identified ChIP binding sites.[39] All 7 of these genes are down-regulated in *Ctsg-PML-RARA* mice. In the SAM analysis (fold change ≥ 2 and a FDR = 0), there were 21 unique genes that were significantly dysregulated in the *Ctsg-PML-RARA* mice, and 9/21 (43%) are down-regulated in the *Ctsg-PML-RARA* mice.

In the comparison of gene expression between *Ctsg-PML-RARA* and WT GMPs, there were 151 unique, annotated genes that are significantly dysregulated by ANOVA with a p-value < 0.001 and fold change ≥ 2. Of these genes, 104/151 are down-regulated (69%). Only 11/151 GMP ANOVA (7%) hits overlapped with the previously identified ChIP binding sites.[39] 9/11 of these genes are down-regulated in *Ctsg-PML-RARA* mice. In the SAM analysis (fold change ≥ 2 and a FDR = 0), there were 20 unique genes that were significantly dysregulated in the *Ctsg-PML-RARA* mice, and 17/20 (85%) are down-regulated in the *Ctsg-PML-RARA* mice.

In the comparison of gene expression between *Ctsg-PML-RARA* and WT MEPs, there are 28 unique genes that were significantly dysregulated by ANOVA with a p-value < 0.001 and fold change ≥ 2. Of these genes, 15/28 are down-regulated (54%). 2/28 MEP ANOVA (7%) hits overlapped with the previously identified ChIP binding sites.[39] Both of these genes are up-regulated in *Ctsg-PML-RARA* mice. In the SAM analysis (fold change ≥ 2 and a FDR = 0), there were 5 unique genes that were significantly dysregulated in the *Ctsg-PML-RARA* mice, and all 5 are down-regulated in the *Ctsg-PML-RARA* mice.

In the comparison of gene expression between *Ctsg-PML-RARA* and WT promyelocytes, there are 679 unique genes that were significantly dysregulated by ANOVA with a p-value < 0.001 and fold change ≥ 2. Of these genes, 9/679 are down-regulated (1.3%). 20/679 promyelocyte ANOVA hits (3%) overlapped with the previously identified ChIP binding sites.[39] All 20 of these genes are up-regulated in mCG-PR mice. In the SAM analysis (fold change ≥ 2 and a FDR = 0), there were 134 unique genes that were significantly dysregulated in the *Ctsg-PML-RARA* mice, and 1/134 (<1%)) are down-regulated in the *Ctsg-PML-RARA* mice.
